# Neural underpinnings of response inhibition in substance use disorders: weak meta-analytic evidence for a widely used construct

**DOI:** 10.1007/s00213-023-06498-1

**Published:** 2023-11-21

**Authors:** Maximilian Fascher, Sandra Nowaczynski, Carolin Spindler, Tilo Strobach, Markus Muehlhan

**Affiliations:** 1 Department of Psychology, Faculty of Human Sciences, Medical School Hamburg, Am Kaiserkai 1, 20457 Hamburg, Germany; 2 Medical School Hamburg, ICAN Institute for Cognitive and Affective Neuroscience, Am Kaiserkai 1, 20457 Hamburg, Germany; 3 Department of Addiction Medicine, Carl‑Friedrich‑Flemming‑Clinic, Helios Medical Center Schwerin, Schwerin, Germany

**Keywords:** Substance use disorder, Response inhibition, Functional magnetic resonance imaging, Meta-analysis, Inhibitory control

## Abstract

**Rationale:**

Substance use disorders (SUDs) rank among the most severely debilitating psychiatric conditions. Among others, decreased response inhibition capacities could make it more difficult for patients to abstain from drug use and maintain abstinence. However, meta-analyses on the neural basis of response inhibition in SUDs yielded conflicting results.

**Objective:**

In this study, we revisited the neuroimaging research field and summarized the existing fMRI literature on overt response inhibition (Go/NoGo and stop-signal paradigms) across different SUDs.

**Methods:**

We performed a systematic literature review and an activation likelihood estimation (ALE) meta-analysis to investigate the actual convergence of functional deviations observed in SUD samples. Results were further supplied by consecutive robustness measures and a post-hoc random-effects meta-analysis of behavioural data.

**Results:**

We identified *k* = 21 eligible studies for our analysis. The ALE analysis indicated a significant cluster of convergence with its statistical peak in the right anterior insula. Consecutive analyses, however, indicated this result was not robust and susceptible towards publication bias. Additionally, a post-hoc random effects meta-analysis of the behavioural parameters of Go/NoGo and stop-signal paradigms reported by the included studies revealed no significant differences in task performance comparing SUD samples and controls.

**Conclusion:**

We discuss that the role of task-based response inhibition may require some refinement as an overarching marker for SUD pathology. Finally, we give a few prospects for future research that should be further explored in this context.

**Supplementary Information:**

The online version contains supplementary material available at 10.1007/s00213-023-06498-1.

## Introduction

Substance use disorders (SUDs) list among the most serious mental illnesses. Lifetime prevalence ranges from 8.0 to 17.5% for alcohol-associated disorders and 1.8 to 3.0% for disorders associated with the use of illicit substances mainly in industrialised countries (Kessler et al. 2004, 2007; Hasin et al. 2007; Merikangas and McClair 2012; Geschwind and Flint 2015). Purposes of consumption include social drinking, satisfaction of curiosity, recreational use, and maladaptive strategies of emotion regulation (Parks and Kennedy 2004; Terry-McElrath et al. 2009).

Aside from considerable diversity in incentives to initiate substance use, all SUDs that require treatment share that they come along with a severe craving for the respective substance as well as a deficient ability to resist consumption despite long-term harmful effects (American Psychiatric Association 2013; MacCoun 2013; Compton et al. 2013; Sayette 2016). These core symptoms of SUD promoted the rationale that inhibition might serve as a promising prospect to be further investigated in SUD patients as a common factor of disease. Inhibition can be defined as the ability to suppress automated or prepotent responding tendencies that are no longer adaptive in a given situation (Nigg 2000; Miyake et al. 2000; Liddle et al. 2001; Aron 2007; Chambers et al. 2009). Impaired capacity for behavioural inhibition is of clinical importance, as it is associated with an increased likelihood of developing and maintaining pathological drug use in SUDs, as well as with a poor prognosis for treatment (Müller et al. 2008; de Wit 2009; Bakhshani 2014; Stevens et al. 2014). Recent findings suggest that patients with SUDs compared to unaffected controls show poorer task-related performances in paradigms measuring response inhibition (Groman et al. 2009; Elton et al. 2014; Smith et al. 2014; Weafer et al. 2014; Morris et al. 2016; Byrne and Worthy 2019).

The latest data-driven meta-analyses on response inhibition aberrations in SUDs, however, show inconsistent findings. Le et al. (2021) demonstrated reduced activity in the dorsal anterior cingulate and middle frontal gyrus in non-abstinent samples with SUD compared to controls. The authors did not report a contrast independent of consumption status. In contrary, the meta-analysis by Qiu and Wang (2021) shows that aberrations in SUDs were expressed by reduced activity in areas of the inferior frontal gyrus (IFG), supramarginal gyrus, temporal areas, and insula but showed increased activity in the cerebellum. It seems astonishing that both meta-analyses report considerably different results, although they used the same meta-analytical algorithm (ES-SDM; Radua et al. 2012).

However, previous meta-analyses both included Go/NoGo, Stop-Signal as well as Stroop tasks (Stroop 1935) as response inhibition measures in their analysis. The Go/NoGo task (GNGT; Donders 1969; Verbruggen and Logan 2008) and the Stop-Signal task (SST; Logan et al. 1984) both require the repeated execution of a motor response to presented Go-stimuli. However, next to Go-stimuli, NoGo- or Stop-stimuli are presented over the course of trials during the GNGT and the SST, respectively. On these stimuli, the subjects are instructed to refrain from responding which demands the subject to inhibit prepotent motor tendencies. Similar to a variety of other cognitive tasks subsumed as incongruency tasks (Cieslik et al. 2015) which also require supressing a response in incongruent trials, there is strong indication that these are associated with other cognitive functions exceeding the requirements to inhibit a prepotent response only (interference resolution, distractor resistance, etc.). The perception of GNGT and SST as particularly relevant measures concerning SUDs may be due to their exclusive focus on inhibiting overt behavioural responses, which could translate into the ability to stop continuous drug use. The predominance of inhibition training in SUDs following a GNGT or SST scheme favours perceiving these tasks as particularly relevant, whereas to the best of our knowledge such therapeutic regimes for Stroop tasks have not been designed (Verdejo-Garcia 2016; Verdejo-Garcia et al. 2023).

Debating Stroop interference as sufficiently different from GNGT and SST response inhibition is also supported by neuroimaging meta-analyses. When investigated in parallel, these studies demonstrate that separating response inhibition (GNGT and SST) and cognitive inhibition (Stroop) in two taxonomic divisions accrued due to considerably distinctive recruitment of neuronal networks and participating areas (e.g. lacking basal ganglia involvement in Stroop tasks) (Zhang et al. 2017; Hung et al. 2018; Rodríguez-Nieto et al. 2022). In particular, Zhang et al. (2017) were able to show that interference resolution shows stronger associations with the ventral attention network (VAN) than response inhibition (GNGT & SST), whereas response inhibition can be assigned more to the fronto-parietal network (FPN). At the same time, both GNGT and SST place more converging demands on brain areas involved following their execution (Chambers et al. 2009; Swick et al. 2011; Sebastian et al. 2013; Zhang et al. 2017; Raud et al. 2020).

Based on these considerations, the understanding of our work is to exclude the Stroop task from our meta-analysis due to its entanglement in interference and conflict resolution. We therefore reduce data eligible to a narrower notion of response inhibition by only including GNGT and SST paradigms as an embodiment of response inhibition. Thus, we aim to re-examine the field of altered neural signatures of response inhibition in SUDs to negotiate conflicting findings of previous works with our meta-analysis by utilizing the Activation-Likelihood-Estimation approach (ALE; Laird et al. 2009; Eickhoff et al. 2012) testing for actual convergence of neuroimaging results in this regard. ALE makes use of the idea to find the most likely spatial convergence of activation patterns that are reported across studies and that false-positive results should not be replicable. It accounts for the spatial uncertainty of fMRI measurements by treating each statistical peak of an activation event as a Gaussian distribution function of activation likelihood. This approach diverges from modelling the significance of a finding based on effect sizes as previous studies did. We have further supplied our results by consecutive analyses. First, we analysed these ALE-derived clusters with a behavioural characterization and paradigm class analysis method to provide further insight into cluster co-activation across behavioural experiments and experimental tasks. Second, we performed a meta-analytic connectivity modelling (MACM) approach to investigate whether convergence clusters exhibit co-activation patterns with larger brain networks and consecutively calculated the robustness of the results towards publication bias using noise data. Third, we performed a post-hoc meta-analysis of behavioural measures reported across GNGT and SST experiments comparing SUD samples and controls.

## Methods

### Search strategy and data acquisition

For the literature research, we followed the PRISMA guidelines and state-of-the-art guidelines for neuroimaging meta-analysis (Müller et al. 2018; Tahmasian et al. 2019; Page et al. 2021). We preregistered our meta-analysis using PROSPERO (https://www.crd.york.ac.uk/prospero/display_record.php?ID=CRD42022374754). Aside the consideration of the PRISMA guidelines, adherence to the guidelines of coordinates-based meta-analyses is essential to generate robust and unbiased findings. The guidelines ensure that the results are adequately powered and can be confidently interpreted by meeting all the assumptions of the applied meta-analytic algorithm and that the results are generalisable. Checklists for PRISMA-guidelines and for neuroimaging meta-analyses recommendations can be obtained through Table S1 and Table S2 in the supplement, respectively. In the following, we report on the literature search inclusion and exclusion criteria. For the literature search, we used EBSCOhost (https://search.ebscohost.com/) which includes a wide range of databases such as PsycINFO, PsycARTICLES, Medline Complete, CINAHL Complete, and Psychology and Behavioral Sciences Collection databases. Furthermore, we also extended our search to the PubMed database. Due to the revision of diagnostic taxonomies regarding SUDs which have been introduced by DSM-5, disorder classifications deviate from previous versions of the DSM (Jones et al. 2012; American Psychiatric Association 2013). Yet, we also considered studies that still refer to the recently omitted categories of ‘substance dependence’, ‘substance abuse’, and ‘harmful use’. We then formulated some necessary inclusion criteria: (1) Only studies that are written in English and peer-reviewed were used for our review. (2) Since this type of meta-analysis is based on coordinates that must be comparable, only those studies that reported coordinates in standardized reference space (Talairach or MNI) were used. (3) All contrasts used in this analysis must contain a population with SUDs and compare it with a sample of control participants. Control groups did not meet criteria for psychiatric disorders and also had no history of problematic substance use, no SUD diagnosis, or scored in a normative range for SUD-relevant self-report instruments (e.g. AUDIT). The following characteristics led to exclusion: First, there are a few methodological limitations to the admissibility of studies, which we list successively. (1) Studies that are not primary studies, but any review articles are inappropriate for a coordinates-based meta-analysis and were not considered. (2) For this form of data integration, we only considered studies reporting whole-brain fMRI measurements. We excluded explicit and hidden region-of-interest (ROI) studies, as they narrow the focus to brain regions that have been pre-selected by researchers in the first place. ROI studies face the limitation that true effects at the whole-brain level could be overseen since statistical testing is limited to a given pre-defined volume. Instead, it offers a statistical advantage, as the number of multiple comparisons is significantly reduced and is therefore more suitable for hypothesis-driven comparisons of isolated brain volumes. Regarding ALE, the inclusion of ROI-based comparisons nevertheless would create a statistical bias in favour of some high-frequently investigated brain areas for which it is uncertain whether they would survive correction for multiple comparisons on the whole-brain level. (3) Studies were only considered if they recruited at least *n* ≥ 10 participants per group to ensure minimum power of comparisons. (4) Since null findings do not allow any spatial allocation of coordinates, they cannot be meaningfully integrated in an ALE meta-analysis (Eickhoff et al. 2012). The same applies to studies that do not make coordinates accessible. We will describe how we accounted for this confirmation tendency of our analysis in a later paragraph within the method section (see Methods paragraph ‘Fail-Safe-N’ (FSN)).

Furthermore, we limited the inclusion of studies for our analysis by a few sample-related boundaries. (5) Children and adolescent samples (aged < 18) were not included due to anatomical incomparability to adults and deviating developmental stages. (6) Comorbid conditions have been regularly described in psychiatric populations (Wittchen 1996; Lépine et al. 2005; Kessler et al. 2007). Comorbidities with depressive disorders, ADHD or personality, and trauma-associated disorders can be found almost regularly and are often underreported. SUDs can therefore rarely be examined in isolation. However, due to considerable differences in functional architecture, populations with severe comorbidities such as psychotic or delusional disorders were not included. Since we examined a wide range of different SUDs in our analysis, we decided to include samples who show polysubstance use and polytoxicomanic behaviours. Moreover, comorbid tobacco use is observable in almost every SUD population (Anthony et al. 1997; Bobo and Husten 2000; Subramaniam et al. 2016). We applied the following search mask: ((“alcohol*” OR “tobacco” OR “nicotine” OR “smok*” OR “cannabi*” OR “marijuana” OR “thc” OR “cocaine” OR “amphetamine*” OR “methamphetamine” OR “stimulant*” OR “ecstasy” OR “mdma” OR “opiate*” OR “morphine” OR “heroin” OR “benzodiazepine*” OR “analgetic*” OR “hallucinogen*” OR “lsd” OR “ketamine” OR “fentanyl” OR “drug*” OR “substance”) AND (“functional magnetic resonance imaging” OR “fmri” OR “functional MRI”) AND (“response inhibition” OR “go nogo” OR “stop signal”)).

### Activation likelihood estimation (ALE) meta-analysis

ALE models the spatial uncertainty of the extracted coordinates of functional alterations using a 3D-Gaussian function (Eickhoff et al. 2009, 2012; Laird et al. 2009; Turkeltaub et al. 2012). It thus takes account of the spatial uncertainty of highly processed fMRI data. Small sample studies are contemplated with a correspondingly higher spatial uncertainty as reported statistical maxima of coordinates are less likely to be sufficiently precise, whereas larger studies should demonstrate more reliable effects resulting in a less spatially broad 3D-Gaussian function. Therefore, probability maps of the reported local maxima of coordinates were built and tested against the null hypothesis of a random spatial distribution aiming to find the most likely spatial convergence of activation patterns observed across contributing studies. We used BrainMap GingerALE v3.0.2 (http://brainmap.org) for our meta-analysis. Since there were more studies that reported their results in the Montreal Neurologic Institute (MNI) reference space than those that chose the Talairach space (see the “Results” section), the latter were transferred to the MNI space using the Lancaster transformation that is implemented in abovementioned used software (Lancaster et al. 2007; Laird et al. 2010), thus keeping the transfer costs as low as possible. This allows the acquisition of an integrated density function of above-chance convergence in the human brain (Eickhoff et al. 2009; Laird et al. 2011a). Following this procedure, we subjected these accumulated ALE values to further statistical testing: ALE values can be converted to p-values to identify regions that withstand testing against a randomly generated empirical null distribution. We tested against 1000 permutations based on the identical number of extracted foci, contrasts, and subjects using these randomly generated datasets. Due to a considerable number of voxels being tested against the null hypothesis of random convergence, it is crucial to correct for multiple testing to prevent from accumulations of type-I alpha errors (Laird et al. 2005; Eickhoff et al. 2012). We accounted for this by setting cluster-level family-wise-error correction (cFWE) with *p* < 0.05 for the cluster-forming threshold and *p* < 0.001 for the voxel-wise threshold (Eickhoff et al. 2016; Flandin and Friston 2019) as this is the gold-standard among correction methods within ALE (Frahm et al. 2022). Before quantitative integration, we checked any mask outliers for plausibility.

### Behavioural characterization and paradigm class analysis

Any ALE clusters identified in this way were subjected to a behavioural domain analysis and a paradigm class analysis in a subsequent step. We performed these analyses with the respective tools provided by Mango v4.1 (http://ric.uthscsa.edu/mango/; behavioural domain v3.1; paradigm analysis v1.6) provided by Lancaster et al. (Lancaster et al. 2012). Significant ALE clusters were then masked as ROIs and underwent comparison with the vast metadata of thousands of fMRI studies provided by BrainMap database. This further leads to a characterization of behavioural or task-related processes in which the activation of a respective cluster has been involved in previous experiments (Laird et al. 2011b). The domains of the behavioural domain analysis are divided into the superordinate categories action, perception, cognition, emotion, and interoception, in which 60 subcategories can be identified. The paradigm class analysis draws information from data including 111 different experimental paradigms investigated in fMRI. Depending on the cluster characteristics, it is feasible to create a profile of behavioural domains for a specific cluster and investigate the clusters relevance to certain experimental paradigms. To conduct this analysis, the masks had to be transformed into Talairach reference space. Regarding these analyses, it is fundamental to apply a more conservative correction for multiple testing (Bonferroni-correction-alike). Consequently, we classify the behavioural domain and paradigm class analyses as significant if they exceed a threshold of *z* = 3.0 (with *p* < 0.05) and *z* = 3.3 (with *p* < 0.05), respectively (Lancaster et al. 2012). For further information, see http://ric.uthscsa.edu/mango/versionhistory.html#v401.

### Meta-analytic connectivity modelling (MACM)

MACM is a valuable method for investigating how ALE-derived clusters are functionally organized within larger brain networks (Eickhoff et al. 2011; Fox et al. 2014; Langner et al. 2014). The cluster is taken as the ROI in an analysis measuring co-activation of spatially separated neural activation patterns using the vast fMRI data available in the BrainMap database. MACM has been shown to be a reasonable network co-activation estimator analogous to resting-state functional connectivity (Robinson et al. 2012). Using Mango v4.1, we masked significant clusters of convergence as ROIs and transferred them into the Talairach reference space. This mask has been entered into Sleuth and can be compared with the database. As searching filters for studies of co-activation, we applied “Diagnosis: Normals”, “Context: Normal Mapping”, and “Activations: Activations Only”. The acquired co-activated coordinates from a plethora of fMRI studies were saved as a coordinate file and applied to GingerALE in the same way as we perform the main ALE analysis, using identical thresholding. The results then show distinct neural networks based on convergence resulted from observed co-activated sights across the brain.

### Fail-safe-N (FSN)

A central limitation of ALE analyses is that they are not sensitive to publication bias and vulnerable to unilateral confirmation tendencies. Samartsidis et al. (2020) estimate that in neuroimaging, approximately 6–30% of studies are not published because they show negative or null results, an effect known as the “file drawer problem”. To estimate robustness of our results against this type of publications bias, we performed a fail-safe-N (FSN) calculation explicitly adapted to ALE meta-analyses by Acar et al. (2018). First, we defined the critical lower bound of the FSN below which the data cannot be considered robust, because publication bias can be assumed. According to Samartsidis et al. (2020), we conservatively set this threshold at 30%, which corresponds to seven studies. Noise studies were then generated which matched the original included studies in sample size and number of foci, but in which the foci were randomly distributed across the brain. The FSN calculation is an iterative procedure that starts with the number of noise studies specified for the lower boundary. The noise studies were added to the original dataset, and the ALE meta-analysis was repeated. Then, noise studies were added successively until the cluster under consideration is no longer significant and the FSN is reached. Noise studies were generated using R Studio v4.1.0 following the algorithm of Acar and colleagues (2018) (https://github.com/NeuroStat/FailSafeN).

### Post-hoc exploratory meta-analysis of behavioural data

When seeing through the included studies, it was noticeable that only some studies report an inferior task performance in SUD samples compared to the respective control groups on task relevant measures. Therefore, we performed a post-hoc random-effects meta-analysis of the most frequently reported behavioural data (commission errors, CE; omission errors, OE; Go-reaction time, Go-RT; and stop-signal reaction time, SSRT). As some studies reported CE and others reported NoGo-accuracy, we treated NoGo-accuracy as the inverse of CE and pooled these data into one estimator, which we subsume as CE in the following sections. The rationale behind this is that a lower NoGo-accuracy should logically be accompanied by an increased rate of CEs. This was done in an analogous way for the OEs. We refrained from calculating further usual meta-analytic metrics such as publication bias estimates, since our systematic literature search was not designed to analyse behavioural data initially. We fitted a random-effects model with the effect size hedge’s *g* for standardized mean differences wherever data were available and *tau*^2^ as well as *I*^2^ as estimates of heterogeneity. All analyses were performed using R statistics (R Core Team 2021) with the package *metafor* (Viechtbauer 2010).

## Results

**Fig. 1:**
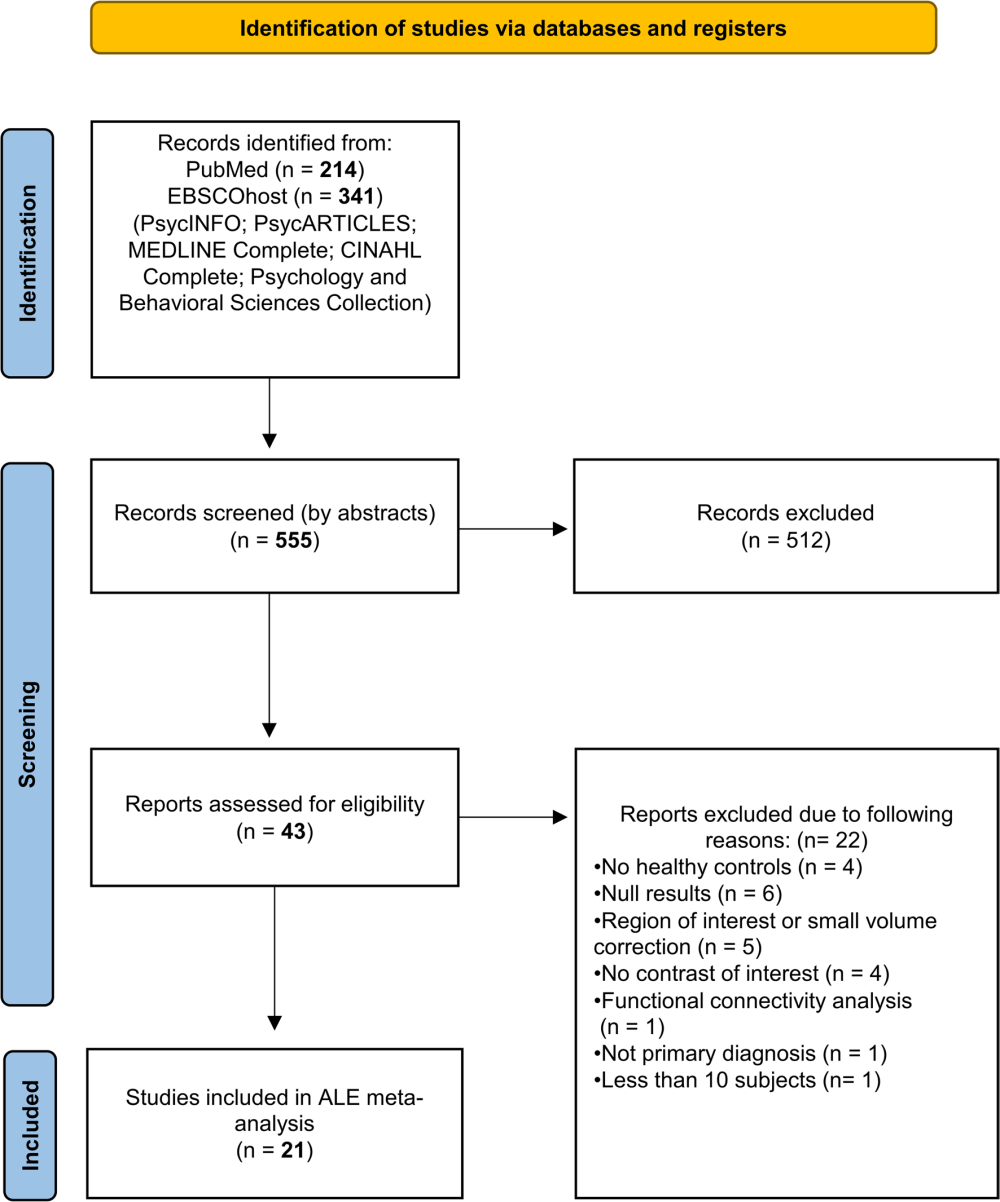
Prisma flow diagram displaying the procedure of the systematic literature search (PRISMA-statement; Page et al. 2021)

**Fig. 2:**
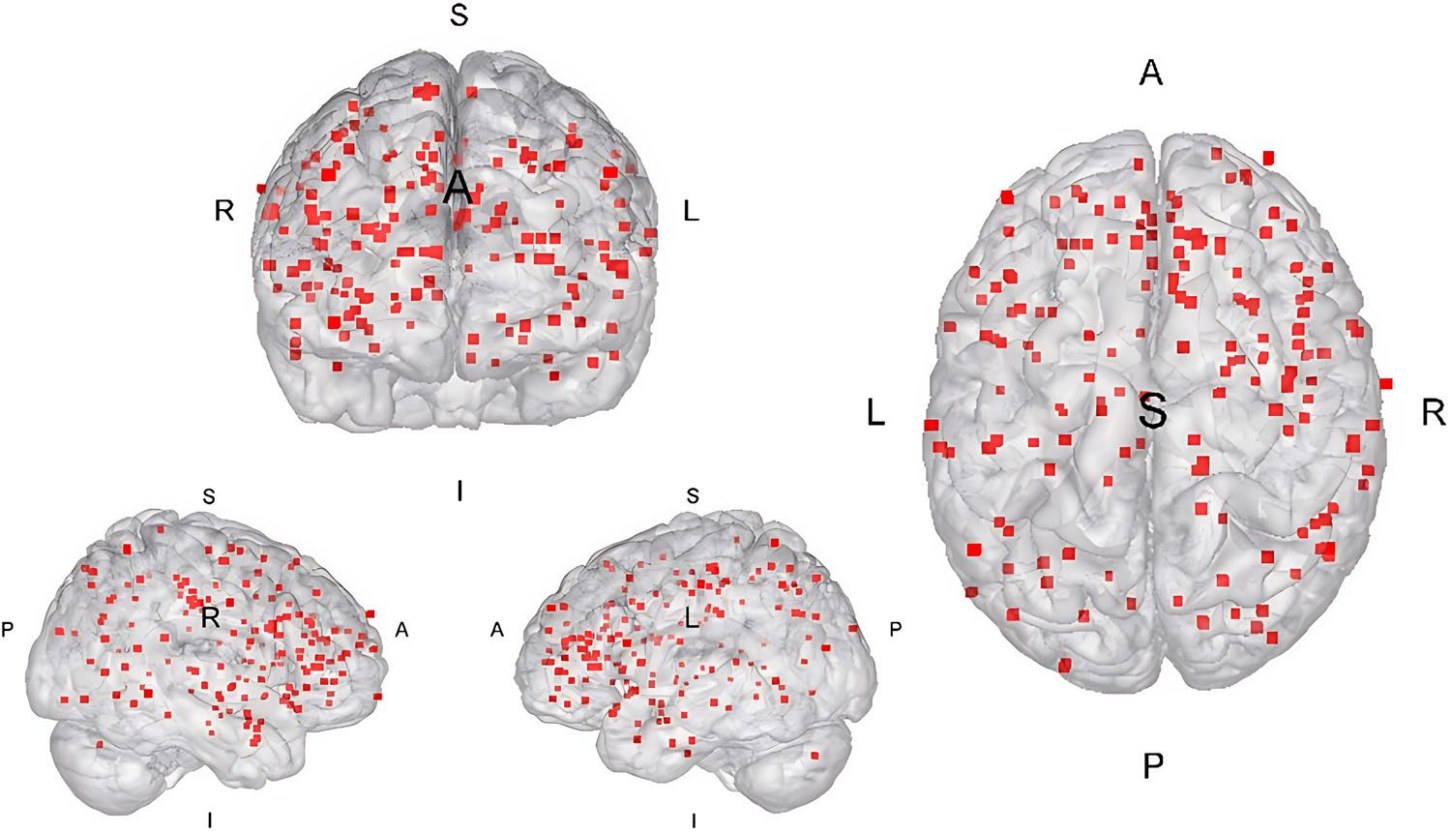
Foci-distribution of experiments integrated in the ALE meta-analysis. Every red data point represents a peak coordinate (foci) of included studies. Foci are displayed on a MNI152 reference space glass brain surface (Colin27_T1_seg_MNI template). A anterior; L left; P posterior; R right; S superior. Mango v4.1 was used to create the image. (http://ric.uthsc sa.edu/mango/)

We identified *k* = 21 studies yielding *k* = 22 experiments eligible for inclusion and meta-analytic integration, containing *n* = 538 participants with SUD and 163 peak coordinates reported across the brain. Different phases of the literature search can be obtained via Fig. 1. We did not suspect any overlap between the studies because the studies with the same authors differed in terms of the sample studied, the collection parameters, the study design, and the demographic characteristics. The study by Gerhardt et al. (2021) investigated both GNGT and SST paradigm in the same sample. We have treated these data as a single experiment because splitting the experiments into two different ones would suggest statistical independence, which we argue is not given by using the same sample. Furthermore, in this study we suspected errors in 2 peak coordinates that were outside the mask when first checked or incongruent with the brain location declared. The first coordinate (*x* =  − 38, *y* = 72, *z* = 44) should indicate a peak in the inferior parietal lobule, whereas this is located far outside the mask (Gerhardt et al. 2021). The sign of the *y*-coordinate seems to be incorrect, so that we assume, in line with the reported brain area, that the coordinate should have been *x* = 38, *y* =  − 72, *z* = 44. Similarly, we suspect another error regarding the coordinate *x* =  − 4, *y* = 12, *z* =  − 26. The coordinate label denotes a sight in the temporal pole, so we assume the coordinate should be *x* =  − 40, *y* = 12, *z* =  − 26 which would then correspond to the respective labelling. After adjusting the coordinate to the latter version, we integrated the coordinate into the analysis. The inclusion of the *k* = 21 studies led to a composition of SUD samples with alcohol use disorder being most prevalent (42%), followed by stimulants (28%), tobacco (24%), and heroin (5%). A total of *k* = 16 studies investigated response inhibition using the GNGT and *k* = 5 studies used SST in this regard. Study characteristics containing information regarding demographics and experimental design can be obtained through Table 1 whereas Fig. 2 displays foci distribution of contributing experiments.

**Table 1:** Demographic and clinical sample characteristics of the included studies

	Sample size, *N* (% female)		Mean age, *M* (SD)									
Study	SUD	CON	SUD	CON	Substance	Illness duration years, *M* (SD)	Abstinence days	Task	NoGo/stop ratio	Contrasts	Corrections	Foci
Ahmadi et al. (2013)	56 (66%)	36 (45%)	19.0 (0.5)	18.8 (1.0)	Alcohol	n.a	n.a	GNGT	15%	successful no go > go	FDR* p* < 0.025, cluster extent* k* ≥ 25	11
Alderson Myers et al. (2021)	19 (57%)	18 (50%)	23.5 (3.1)	25.6 (4.1)	Alcohol	n.a	n.a	GNGT	17%	error no go > successful nogo	FDR* p* < 0.05, cluster-level* p* = 0.05, cluster extent* k* ≥ 29	3
Ames et al. (2014)	21 (52%)	20 (65%)	20.2 (1.4)	20.8 (1.0)	Alcohol	n.a	n.a	GNGT	20%	no go > go	Uncorrected* p* < 0.001	3
Campanella et al. (2017)	19 (59%)	17 (63%)	24.7 (3.0)	25.8 (4.2)	Alcohol	n.a	1	GNGT	30%	error no go > correct nogo	cFWE* p* < 0.05, cluster-level* k* ≥ 696	6
Ceceli et al. (2023)	26 (15%)	26 (19%)	44.1 (8.2)	42.7 (7.1)	Cocaine	16.0 (8.2)	n.a	SST	33%	successful stop > stop error	Voxel-level* p* < 0.001, cluster-level* p* < 0.05	2
Chaarani et al. (2019)	15 (22%)	15 (37%)	24.8 (5.8)	22.6 (1.5)	Tobacco	n.a	1	SST	16%	successful stop > stop error	Voxel-level* p* < 0.001, cluster-level* p* < 0.05	4
Czapla et al. (2017)	19 (10%)	21 (19%)	51.2 (7.4)	41.9 (9.9)	Alcohol	11.5 (8.5)	21	GNGT	20%	no go > go	cFWE* p* < 0.05, cluster extent* k* ≥ 696	6
Fu et al. (2008)	30 (0%)	18 (0%)	33.4 (6.0)	29.6 (6.9)	Heroin	6.3 (3.5)	53.5	GNGT	25%	no go > go	Voxel-level* p* < 0.05	22
Gerhardt et al. (2021) (Exp. 1 = GNGT) (Exp. 2 = SST)	15 (13%)	15 (40%)	47.0 (12.3)	41.9 (14.9)	Alcohol	n.a	19.0	GNGT; SST	1. 13% 2. 13%	1. no go > go 2. stop > go	cFWE* p* < 0.05, cluster threshold* p* < 0.01 (*k* ≥ 460)	8; 2
Grieder et al. (2022)	45 (56%)	25 (54%)	43.6 (10.4)	43.1 (8.2)	Alcohol	12.6 (11.9)	84.0	GNGT	15%	nogo > go	Uncorrected* p* < 0.001, cluster threshold *k* = 10	19
Hester (2004)	15 (40%)	15 (53%)	40.0 (n.a.)	31.0 (n.a.)	Cocaine	14 (n.a.)	1.7	GNGT	12%	successful no go > go	Cluster corrected* p* < 0.05	3
Hester et al. (2013)	15 (13%)	15 (13%)	38.2 (n.a.)	42.7 (n.a.)	Cocaine	5.1 (n.a.)	335	GNGT	20%	no go > go	Voxel-level* p* < 0.001, cluster extent 142 µl	9
Kalhan et al. (2022)	20 (50%)	20 (50%)	24.3 (4.7)	23.7 (4.3)	Tobacco	n.a	< 1	SST	25%	successful stop > go	cFWE* p* < 0.05	12
Kaufman et al. (2003)	13 (38%)	14 (71%)	37.0 (4.5)	30.0 (8.7)	Cocaine	n.a	< 1	GNGT	6%	no go > go	Voxel-level *p* < 0.05 (simulated)	7
Li et al. (2009)	24 (25%)	24 (25%)	38.7 (8.3)	35.5 (5.9)	Alcohol	n.a	> 14	SST	25%	successful stop > stop error	Uncorrected* p* < 0.001, cluster extent k *k* ≥ 5	15
Luijten et al. (2013)	25 (28%)	23 (40%)	22.6 (2.8)	21.7 (1.8)	Tobacco	7.2 (3.0)	< 1	GNGT	12%	successful no go > successful go	Cluster-level* p* < 0.01 (Monte Carlo)	4
Ma et al. (2015)	13 (7%)	10 (30%)	37.4 (5.3)	35.2 (7.3)	Cocaine	n.a	n.a	GNGT	25%	no go > go	cFWE* p* < 0.05	5
Morein-Zamir et al. (2013)	32 (6%)	41 (36%)	34.5 (7.8)	31.7 (8.5)	Stimulants	15.9 (6.7)	< 1	SST	16%	successful stop > stop error	Uncorrected* p* < 0.001, cluster extent* k* ≥ 10	3
Nestor et al. (2011) (Exp. 2)	10 (50%)	13 (38%)	23.0 (1.0)	23.6 (1.3)	Tobacco	6.7 (1.2)	< 1	GNGT	10%	no go > go	Voxel-level* p* < 0.001, cluster-level 328 µl	14
Nestor et al. (2019)	21 (19%)	35 (20%)	46.2 (2.0)	41.1 (1.5)	Alcohol	n.a	428.0 (128.6)	GNGT	12%	no go > go	Cluster-level* p* < 0.05 (Bonferroni)	3
Weywadt et al. (2017)	81 (59%)	38 (71%)	57.0 (1.4)	61.0 (1.4)	Tobacco	37.0 (1.7)	< 1	GNGT	16%	successful no go > go	FDR* p* < 0.01, cluster-level* p* = 0.05	1

*SUD* substance use disorder, *FDR* false-discovery-rate, *cFWE* family-wise error correction, * CON* controls, *M* mean, *n.a.* not available, *SD* standard deviation

### ALE meta-analytic results

**Fig. 3:**
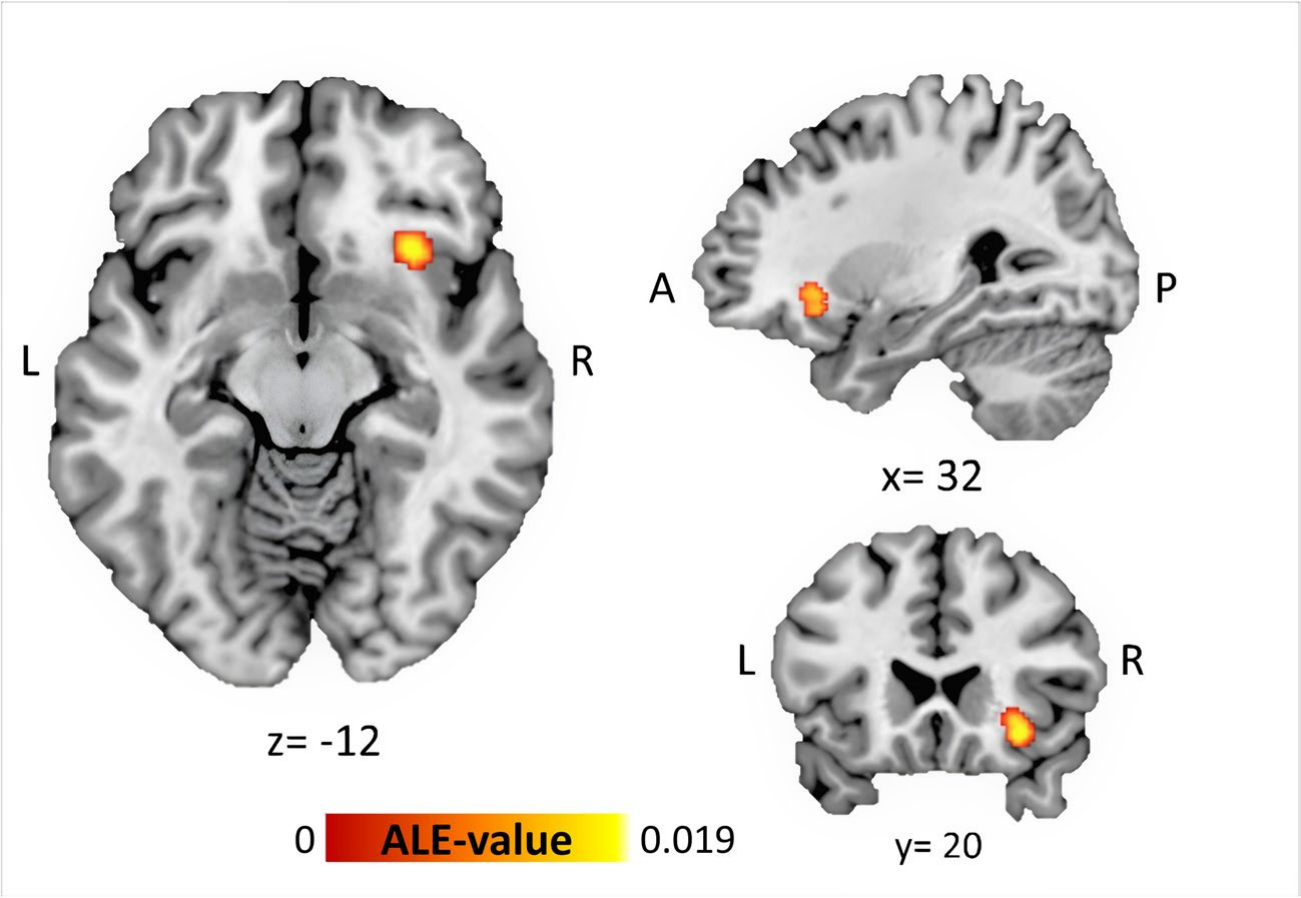
ALE-derived cluster of significant convergent hemodynamic alterations comparing SUD samples and controls during response inhibition tasks. The peak is located at (*x* = 32, *y* = 20, *z* =  − 12) in the rAI. Coordinates refer to MNI152 reference space (Colin27_T1_seg_MNI template). A anterior; L left; P posterior; R right; S superior. Image has been created with Mango v4.1 (http://ric.uthsc sa.edu/mango/)

ALE analysis revealed a cluster with significant convergence of altered activation between SUD samples and controls in the right hemisphere (cFWE *p* < 0.05 corrected for multiple comparisons). The cluster shows a significant peak in the right anterior insula (rAI), where the insula accounts for 31.6% of the cluster volume and extends over the right claustrum and the orbital part of the IFG with 31.6% and 10.5% of the cluster extent, respectively. 26.3% could not be labelled (see Table 2, Fig. 3). Contributing studies (*n* = 3) include samples of alcohol- (Grieder et al. 2022), tobacco- (Nestor et al. 2011), and cocaine-associated (Morein-Zamir et al. 2013) SUDs once each (Table 1). All contributing studies used voxel-wise or cluster-wise thresholds that can be perceived as liberal. Regarding the direction of the effects, one contributing study reported an increased activation in the respective cluster in samples with SUD (Grieder et al. 2022) whereas two studies reported a decreased activation (Nestor et al. 2011; Morein-Zamir et al. 2013). The subsequent behavioural domain and paradigm class analysis showed no significant associations. Our consecutive FSN analysis showed that the significance cluster withstands *n* = 2 (9.5%) noise studies and therefore showed strong susceptibility to potential publication bias. The ALE results thus turn out not to be a very stable finding.

**Table 2:** ALE-derived cluster of significant convergence

	Peak voxel coordinate (MNI)							
Anatomical Label^a^	*x*	*y*	*z*	BA	Cluster Size (mm^3^)	ALE * (10^−2^)^b^	No. of contributing experiments (%)	Fail-Safe-N
R Cerebrum.Sub-lobar.Insula	32	20	− 12	13	800	1.95	3 (14.3%)	2 (9.5%)

*x*, *y*, *z* coordinates are displayed in MNI reference space

*BA* Brodmann area, *R* right

^a^Anatomical labelling refers to MNI atlas (nearest grey matter) of peaking coordinate

^b^Maximum ALE value of the cluster

### MACM results

**Fig. 4:**
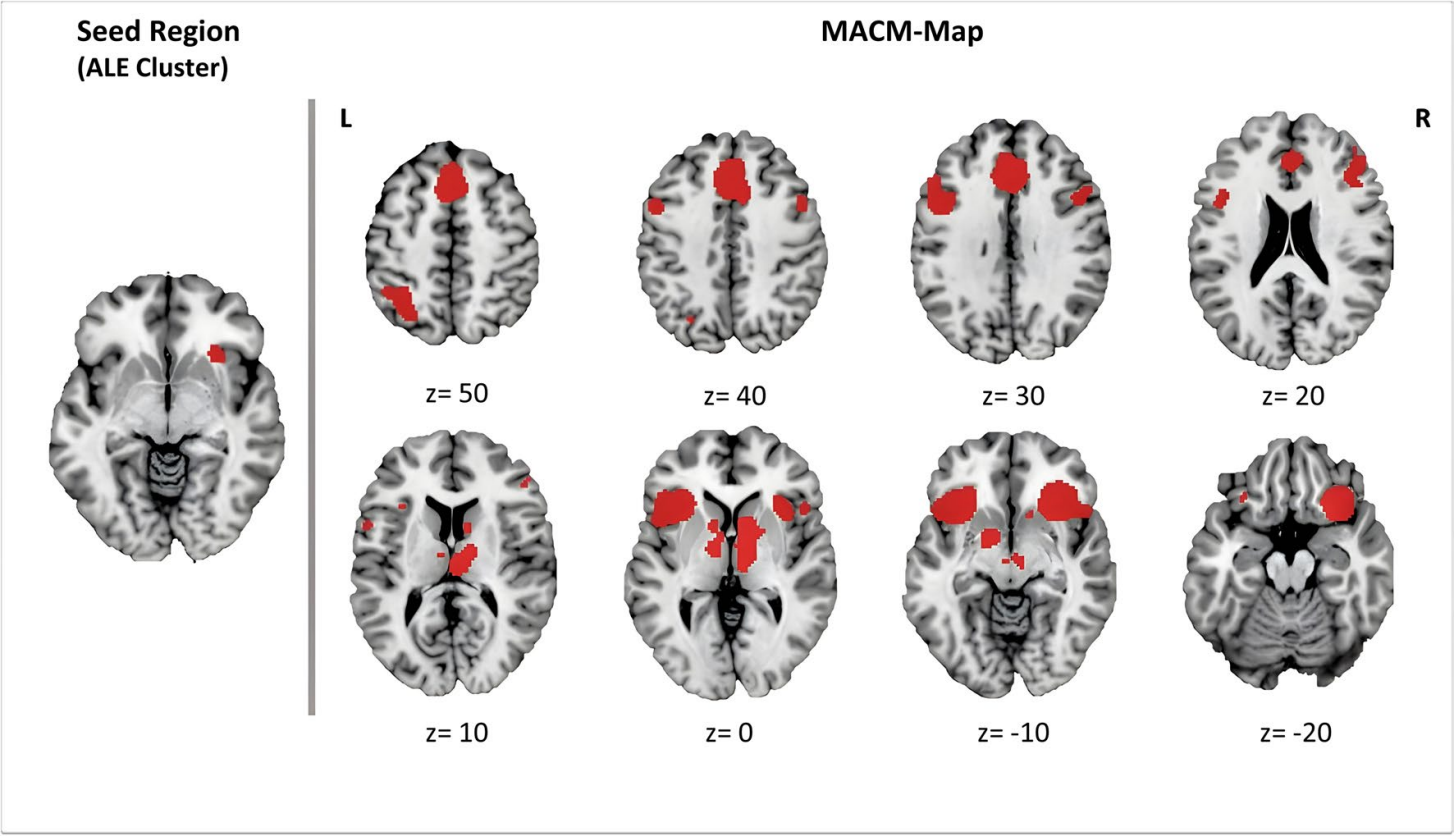
Resulting MACM map. Significant coactivations with the ALE-derived convergence cluster displayed in MNI152 reference space (Colin27_T1_seg_MNI template). L left, R right. Image has been created with Mango v4.1 (http://ric.uthsc sa.edu/mango/)

After the convergence cluster was fed as an ROI mask to the MACM analysis to create a functional connectivity map, the BrainMap database reported coactivations from 137 experiments, with 1939 foci respectively, examining 2115 participants. This allowed 8 clusters (C1-C8) of significant coactivation to be identified (Supplemental Table S1, Fig. 4). These include areas of the cingulate gyrus and medial frontal gyrus (C1), the region around the seed ROI of the rAI (C2) and its contralateral counterpart spanning the left claustrum, left AI and precentral gyrus (C3). Other cortical areas with significant coactivation were observed in bilateral clusters of middle and IFG (C5, C6), as well as left lateral inferior parietal lobule and precuneus (C8). Furthermore, subcortical coactivation was seen in clusters of thalamo-striatal regions extending over the right caudate head and body, the right medial-dorsal and ventral-lateral thalamic nuclei, and all the way to the bilateral mammillary body in the brainstem (C4). Contralaterally, there were significant peaks with nucleus lentiformis in more ventral areas of the striatum (C7).

### Post-hoc exploratory meta-analysis of behavioural data

**Fig. 5:**
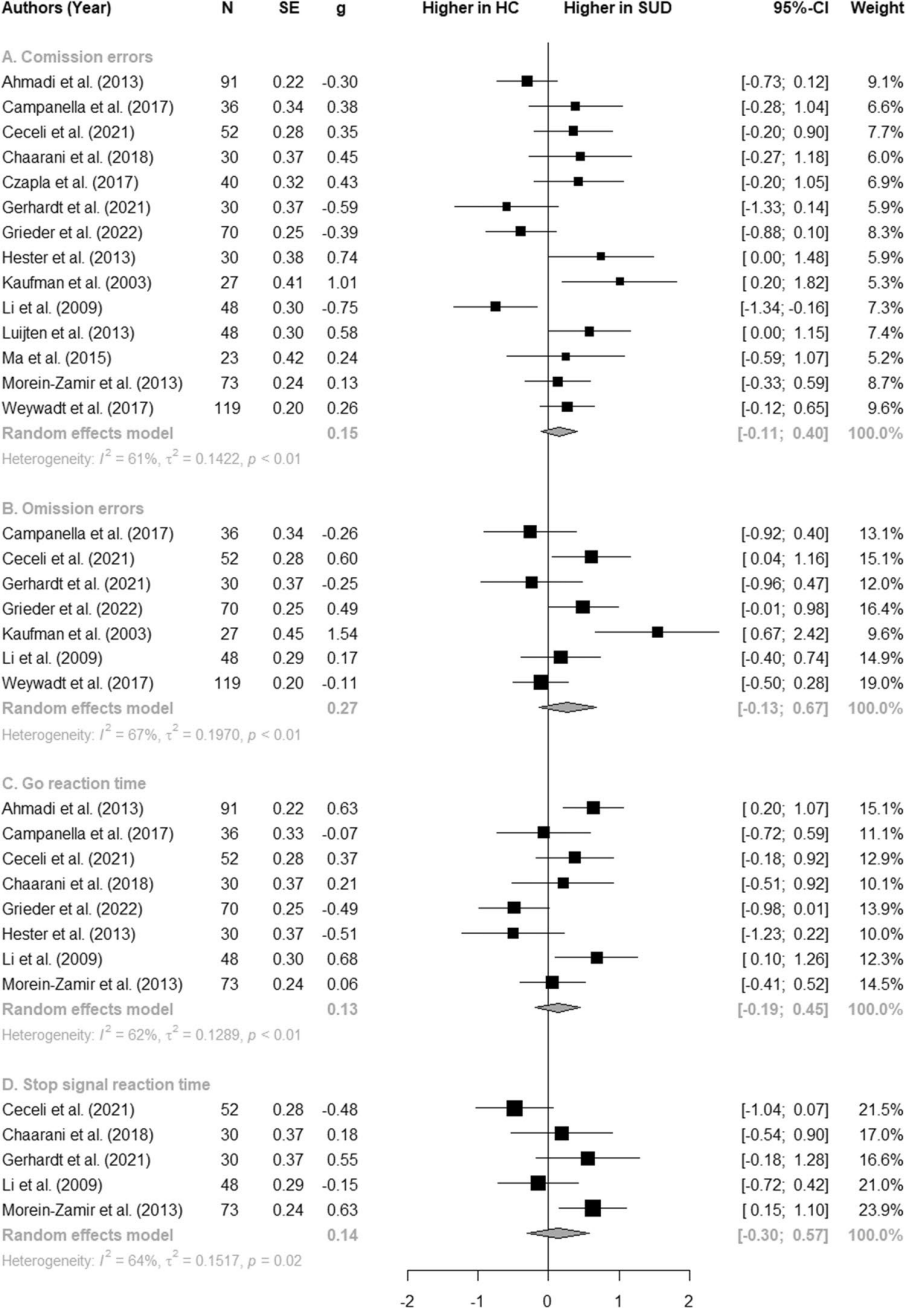
Forest-plot showing meta-analytic computations of behavioural task measures of GNGT and SST

The meta-analysis of behavioural data shows no significant difference in task performance for CE, OE, Go-RT as well as SSRT between SUD samples and controls with effect sizes ranging from *g* = 0.13–0.27 within respective measures. We observed mediocre, but significant, heterogeneity within measures of response inhibition with *I*^2^ ranging from 61 to 67%. Results are displayed in Fig. 5.

## Discussion

With this coordinate-based meta-analysis, we integrated functionally altered haemodynamic response patterns observed in response inhibition tasks (GNGT and SST) comparing SUD samples and control samples. We were able to show a significant convergence cluster that has its statistical peak in the rAI. Behavioural domain and paradigm class analyses yielded no significant associations. The consecutive MACM analysis highlighted a fronto-parieto-striatal circuit to significantly co-activate with the rAI cluster. The FSN analysis of robustness towards publication bias revealed a FSN of *n* = 2, and finally, a subsequent random effects meta-analysis demonstrated no significant differences in any behavioural GNGT or SST measurement between SUD samples and controls.

The rAI is an often-replicated meta-analytically derived area to be involved in the performances of both GNGT and SST (Swick et al. 2011; Criaud and Boulinguez 2013; Sebastian et al. 2013; Zhang et al. 2017; Hung et al. 2018; Puiu et al. 2020). However, the rAI has also been found to be one of the most consistently reported meta-analytic findings of functional aberrations across a vast variety of psychiatric disorders during cognitive and inhibitory control (McTeague et al. 2017; Yan et al. 2022). This suggests that the cluster of convergence we identified might be difficult to disentangle from comorbid conditions. The rAI is an essential node of the salience network (Uddin et al. 2019) involved in a whole range of cognitive functions (Niendam et al. 2012). Recently, the AI has been described as providing a gate keeping function that couples required neuronal resources for relevant tasks facilitating the processing of task-relevant stimuli at an early stage (Molnar-Szakacs and Uddin 2022). In their neuroimaging meta-analysis of response inhibition based on the GNGT, Criaud and Boulinguez (2013) were able to show that rAI functioning is associated with identification properties of complex stimuli rather than sensitivity towards low-frequency NoGo stimuli or working memory load. Thus, it suggests the rAI to support early and basal aspects of salience detection in response inhibition tasks.

Our ALE results partially converge with those of a recent meta-analysis investigating response inhibition related brain alterations in SUDs. Qiu and Wang (2021) found reduced activity in samples with SUD in an area including the rAI and right IFG. Due to certain study overlap in the authors design and ours, this offers a plausible explanation for yielding a similar result in this respect. Nevertheless, their cluster expands to a larger number of voxels in the brain. We were not able to replicate other findings reported by Qui and Wang including reduced activity in the supramarginal gyrus, the middle temporal gyrus and temporal pole or even increased activity of SUD samples in the cerebellum during response inhibition. Some design-related nonconformities may have contributed to the differences. First, their meta-analysis also integrated Stroop tasks as a measure of response inhibition. Our reasoning of response inhibition thus differs from that of previous work but might partly explain that we could not replicate other results. Second, Qiu and Wang used an effect size-based algorithm (ES-SDM; Radua and Mataix-Cols 2009; Radua et al. 2012) for their meta-analytic integration, which differs from the ALE approach in that their computations are based on effect sizes of coordinates and their polarity. In contrast, ALE calculates spatial convergence weighting foci as a function of sample size considering all coordinates irrespective of their effect-size and polarity. An alternative option is that other results are possibly single study driven wherever studies reported high effect-sizes. Remarkably, considering effect size polarity it might be of particular importance in this case. We would assume that since the recently published study by Grieder et al. (2022) showing increased activity rather than reduced activity of the rAI in SUD samples during response inhibition, the outcome cluster using ES-SDM might average out. This suggests that a considerably salient result from this previous meta-analysis may be challenged by an update of eligible data. In the following paragraphs, however, we will discuss why we offer a different conclusion than previous work did, incorporating the results of our subsequent analyses. For although the ALE-derived cluster marks a significant result, relevant measures of robustness and necessary adherence to gold-standard-guidelines intertwined with the validity of this finding must be addressed.

First, the number of studies contributing to the convergence cluster in this meta-analysis is small, with *k* = 3 (14%). Although, after visual inspection of the unthresholded ALE maps, we cannot rule out the possibility that few probability maps reported in other studies may protrude into cluster-containing voxels. The comparison of contributing vs. non-contributing studies did not suggest a noticeable pattern in study characteristics such as the sample examined or experimental design. While contributing studies contained SUD populations, including alcohol, tobacco, and cocaine, we advise the greatest caution in inferring an overarching and consistent disease mechanism in SUDs based on this observation. Moreover, the direction of the effect in the convergence cluster is not uniform. This could make the interpretation of reduced or increased activity in SUD samples in the respective area rather volatile. Second, the FSN we subsequently calculated resulted in *n* = 2, suggesting low robustness towards potential publication bias. Consequently, adding 3 or more noise studies would have led us to no longer identify the cluster as significant, therefore suggesting that not much noise data is in need to provoke a zero distribution. Third, this result would not withstand quality requirements for fMRI meta-analyses (Müller et al. 2018; Tahmasian et al. 2019), as fMRI results of included studies were often corrected too liberally or not corrected. If the criteria for neuroimaging meta-analyses would have been applied strictly, the ALE would have led to a null finding as it would lead to a partially exclusion of now contributing studies’ results. Too liberal or missing corrections for multiple comparisons inflate false positives. Remarkably, despite liberal corrections the results of the eligible studies do not give the impression of overall inflated data since the number of reported foci per study tends to be rather small. A low foci-count per study contrast serves as a further indicator of a high probability of studies remain in the file-drawer (Samartsidis et al. 2020) which is then reinforced by the consideration of the liberal corrections. We must also emphasise that the included studies overall examined small SUD populations, often less than *n* = 20 participants per group. This questions how original studies’ estimators can add up to a convincing meta-analytic result. Considering the evidence level of included studies, some studies with sufficient power might be needed to draw a more confident conclusion. Yet, if all these limitations are now noted, this signifies that the fMRI results of response inhibition alterations in SUDs are less replicable than expected, show clear indicators of susceptibility towards publication bias and, finally, are not in line with state-of-the-art methodological guidelines for coordinate-based meta-analyses.

Besides limited fMRI evidence we report additional results that fuel a reconsideration of response inhibition in SUDs. After initial inspection of behavioural task measures of response inhibition, we observed little or no impairments in many of the SUD samples compared to respective control groups. We decided to analyse them in a subsequent random-effects meta-analysis. Examining the individual task parameters CE, OE, GO-RT and SSRT, we found no significant differences between the SUD populations and those of controls that could suggest an impairment in performance replicated in included fMRI studies. This is in contradiction to other meta-analytical data demonstrating that different SUDs show inferior performances in these task measures (Wright et al. 2014; Smith et al. 2014). Smith et al. (2014) were able to show in their analysis that different substance classes had very different patterns of impairments in these task parameters but did not carry out an analysis of publication bias. Wright et al. (Wright et al. 2014), on the other hand, did not differentiate by substance classes, but were able to demonstrate an equal deficit in CE as well as OE and slower reaction times of small effect size magnitude that are comparable to our results. They had also found evidence of possible publication bias, which, when corrected, resulted in a further reduction of effect sizes. However, they also discussed that the findings of equivalent deficits in CE and OE associated with slower reaction times in SUDs make it difficult to infer a pattern of disinhibited performance in the accomplishment of the respective tasks. We agree that a pattern of behavioural disinhibition should reveal higher commission errors, lower or no omission errors, and faster reaction times, given that participants are compliant to adhere to the tasks. Our results are most comparable to a recent mega-analysis of original patient data, which showed that for GNGT and SST, only 37.5% and 25% of addicted patients (including pathological gambling), respectively, showed any behavioural inferiority at all (Liu et al. 2019). Thus, these meta-analyses of behavioural data seem to have come across similar limitation as our work did. The issues appear in low effect sizes, heterogeneous findings, and an uncertainty about the role of potential publication bias. Of course, we must caveat that our systematic literature review on fMRI studies did not a priori aim to integrate behavioural data and cannot replace a full and systematic research in this regard. Further, not all task parameters were feasible to include in our behavioural data meta-analysis due to being reported inconsistently or without sufficient information for random-effects meta-analyses (e.g., only *p*-values available) thus limiting our inferences. However, we speculate that neuroimaging studies may not face as high hurdles in the publication process as pure behavioural studies if their task measures cannot show a significant group difference. In the case of a null finding, behavioural studies could thus have greater difficulties in submitting their results to scientific discourse, perhaps leading to an uneven ratio of publication bias probability. Yet, finding no clear behavioural deficit in our analysis adds a further explanation for why we have not been able to find substantial evidence for functional aberrations in our ALE analysis. Future work should therefore address publication bias of behavioural measures of response inhibition in SUD, which is now beyond the scope of our review.

To conclude this work, we would like to draw attention to a few aspects that could be considered in future studies. Looking at the evidence on the therapeutic use of response inhibition trainings, they often yield unsatisfactory treatment effect sizes if significant at all (Houben et al. 2011; Bartsch et al. 2016; Cristea et al. 2016; Batschelet et al. 2020; Schenkel et al. 2023; Reichl et al. 2023). Even in non-clinical samples there is no substantial evidence, that inhibition training leads to improvements in new, untrained transfer situations beyond the training situation in a laboratory context (Enge et al. 2014; Strobach et al. 2014; Strobach and Karbach 2016). There is evidence indicating that response inhibition and impulsive behaviours in the everyday life of SUD patients should not be perceived as closely related constructs by default. Response inhibition and trait impulsivity measures often appear uncorrelated (Horn et al. 2003; Aichert et al. 2012; Wilbertz et al. 2014; Šašinka et al. 2023). This becomes particularly significant when we consider that self-reported impulsive behaviours of patients are among the targets for therapeutic strategies to achieve substance abstinence. It needs to be addressed in the future whether response inhibition paradigms performed in laboratory tasks are indeed capable of simulating conflicting affective, cognitive, and motivational states of patients’ daily lives that warrants these trainings to work. However, these approaches are still largely based on the assumption that response inhibition is a rather stable deficit in SUDs. Recent work by Hildebrandt et al. (2023) offers a new perspective on the intricate relationship between response inhibition and SUD symptoms. In their work, they showed that activity in the right IFG during a stop-signal task in SUD participants was associated with SUD problems (e.g. losing control over the consumption, continue using despite impairing their social relations) when statistically controlling for the degree of substance use. In earlier work, they pointed out that both the degree of use and SUD problems usually correlate, but can vary considerably between as well as within subjects, potentially impeding brain-behaviour associations (Hildebrandt et al. 2021). These results also suggest that a closer look may be needed to better understand the neural correlates of altered response inhibition and its clinical significance in SUDs. For example, analysing the subcomponents of inhibition and viewing it as a dynamic multivariate system may be an important step.

We conclude that the evidence to date for response inhibition as an overarching marker of SUDs in relation to fMRI has some significant limitations in terms of replicability, and that the reputation of the construct differs from the overall robustness of the findings that we would have initially expected. With our work we would take the opportunity to stimulate a conceptual refinement of response inhibition in SUDs. More appropriate methodological approaches, e.g. conducting well powered studies and stricter correction thresholds, as well as conceptual expansion, are now crucial to further address this research topic.

### Supplementary Information

Below is the link to the electronic supplementary material.

Supplementary file1 (PDF 238 KB)

## Data Availability

Data supporting the findings of this study are available within the article and its supplementary materials.
