# Supplementary material for: Neural underpinnings of response inhibition in substance use disorders: weak meta-analytic evidence for a widely used construct
Source: Psychopharmacology (Berl). 2023 Nov 21;241(1):1–17. doi: 10.1007/s00213-023-06498-1 (PMC10774166; doi:10.1007/s00213-023-06498-1)
Supplement: Supplementary file 1 — Supplementary file1 (PDF 238 KB) [file 213_2023_6498_MOESM1_ESM.pdf]

Supplemental Material

Neural underpinnings of response inhibition in substance use disorders: Weak meta-analytic evidence for a widely used construct

Maximilian Fascher[1,2], Sandra Nowaczynski[1,3], Carolin Spindler[1,2], Tilo Strobach[1,2], Markus Muehlhan[1,2]

[1] Department of Psychology, Faculty of Human Sciences, Medical School Hamburg, Hamburg Germany

[2] ICAN Institute for Cognitive and Affective Neuroscience, Medical School Hamburg, Germany

[3] Department of Addiction Medicine, Carl-Friedrich-Flemming-Clinic, Helios Medical Center Schwerin,Schwerin, Germany

Correspondence: Maximilian Fascher

Medical School Hamburg
Am Kaiserkai 1
20457 Hamburg, Germany
Phone: +49 40 361 226 40
E-mail: maximilian.fascher@medicalschool-hamburg.de

Table S1 PRISMA-checklist according to Page et al. (2021) for systematic literature reviews and meta-analyses. Note that for ALE meta-analysis not all criteria proved to be feasible.

Section and Topic	Item #	Checklist item	Location where item is reported
**TITLE**			
Title	1	Identify the report as a systematic review.	Title
**ABSTRACT**			
Abstract	2	See the PRISMA 2020 for Abstracts checklist.	Abstract
**INTRODUCTION**			
Rationale	3	Describe the rationale for the review in the context of existing knowledge.	1 Introduction
Objectives	4	Provide an explicit statement of the objective(s) or question(s) the review addresses.	1 Introduction
**METHODS**			
Eligibility criteria	5	Specify the inclusion and exclusion criteria for the review and how studies were grouped for the syntheses.	2.1 Methods
Information sources	6	Specify all databases, registers, websites, organisations, reference lists and other sources searched or consulted to identify studies. Specify the date when each source was last searched or consulted.	2.1 Methods
Search strategy	7	Present the full search strategies for all databases, registers and websites, including any filters and limits used.	2.1 Methods
Selection process	8	Specify the methods used to decide whether a study met the inclusion criteria of the review, including how many reviewers screened each record and each report retrieved, whether they worked independently, and if applicable, details of automation tools used in the process.	2.1 Methods
Data collection process	9	Specify the methods used to collect data from reports, including how many reviewers collected data from each report, whether they worked independently, any processes for obtaining or confirming data from study investigators, and if applicable, details of automation tools used in the process.	Table S3
Data items	10a	List and define all outcomes for which data were sought. Specify whether all results that were compatible with each outcome domain in each study were sought (e.g. for all measures, time points, analyses), and if not, the methods used to decide which results to collect.	2.1 Methods
	10b	List and define all other variables for which data were sought (e.g. participant and intervention characteristics, funding sources). Describe any assumptions made about any missing or unclear information.	2.1 Methods
Study risk of bias assessment	11	Specify the methods used to assess risk of bias in the included studies, including details of the tool(s) used, how many reviewers assessed each study and whether they worked independently, and if applicable, details of automation tools used in the process.	2.5 Methods & Table S3
Effect measures	12	Specify for each outcome the effect measure(s) (e.g. risk ratio, mean difference) used in the synthesis or presentation of results.	2.2 Methods
Synthesis methods	13a	Describe the processes used to decide which studies were eligible for each synthesis (e.g. tabulating the study intervention characteristics and comparing against the planned groups for each synthesis (item #5)).	2.1 Methods
	13b	Describe any methods required to prepare the data for presentation or synthesis, such as handling of missing summary statistics, or data conversions.	2.2 Methods
	13c	Describe any methods used to tabulate or visually display results of individual studies and syntheses.	Figure 2,3,4,5

Section and Topic	Item #	Checklist item	Location where item is reported
	13d	Describe any methods used to synthesize results and provide a rationale for the choice(s). If meta-analysis was performed, describe the model(s), method(s) to identify the presence and extent of statistical heterogeneity, and software package(s) used.	2.2 Methods
	13e	Describe any methods used to explore possible causes of heterogeneity among study results (e.g. subgroup analysis, meta-regression).	2.6 Methods
	13f	Describe any sensitivity analyses conducted to assess robustness of the synthesized results.	-
Reporting bias assessment	14	Describe any methods used to assess risk of bias due to missing results in a synthesis (arising from reporting biases).	2.5 Methods
Certainty assessment	15	Describe any methods used to assess certainty (or confidence) in the body of evidence for an outcome.	2.5 Methods
**RESULTS**			
Study selection	16a	Describe the results of the search and selection process, from the number of records identified in the search to the number of studies included in the review, ideally using a flow diagram.	Figure 1
	16b	Cite studies that might appear to meet the inclusion criteria, but which were excluded, and explain why they were excluded.	-
Study characteristics	17	Cite each included study and present its characteristics.	Table 1
Risk of bias in studies	18	Present assessments of risk of bias for each included study.	-
Results of individual studies	19	For all outcomes, present, for each study: (a) summary statistics for each group (where appropriate) and (b) an effect estimate and its precision (e.g. confidence/credible interval), ideally using structured tables or plots.	-
Results of syntheses	20a	For each synthesis, briefly summarise the characteristics and risk of bias among contributing studies.	-
	20b	Present results of all statistical syntheses conducted. If meta-analysis was done, present for each the summary estimate and its precision (e.g. confidence/credible interval) and measures of statistical heterogeneity. If comparing groups, describe the direction of the effect.	3 Results &Table 2
	20c	Present results of all investigations of possible causes of heterogeneity among study results.	-
	20d	Present results of all sensitivity analyses conducted to assess the robustness of the synthesized results.	-
Reporting biases	21	Present assessments of risk of bias due to missing results (arising from reporting biases) for each synthesis assessed.	Table 2
Certainty of evidence	22	Present assessments of certainty (or confidence) in the body of evidence for each outcome assessed.	Table 2
**DISCUSSION**			
Discussion	23a	Provide a general interpretation of the results in the context of other evidence.	4 Discussion
	23b	Discuss any limitations of the evidence included in the review.	4 Discussion
	23c	Discuss any limitations of the review processes used.	4 Discussion
	23d	Discuss implications of the results for practice, policy, and future research.	4 Discussion
**OTHER INFORMATION**			

Section and Topic	Item #	Checklist item	Location where item is reported
Registration and protocol	24a	Provide registration information for the review, including register name and registration number, or state that the review was not registered.	2.1 Methods
	24b	Indicate where the review protocol can be accessed, or state that a protocol was not prepared.	2.1 Methods
	24c	Describe and explain any amendments to information provided at registration or in the protocol.	-
Support	25	Describe sources of financial or non-financial support for the review, and the role of the funders or sponsors in the review.	-
Competing interests	26	Declare any competing interests of review authors.	„Conflict of interest“‘
Availability of data, code and other materials	27	Report which of the following are publicly available and where they can be found: template data collection forms; data extracted from included studies; data used for all analyses; analytic code; any other materials used in the review.	2.1 Methods & 3.2 Results

Table S2 Neuroimaging meta-analyses checklist formulated by Müller et al. (2018).

**The research question is specifically defined**	✓ We compared fMRI activation patterns during response inhibition between SUD patients and HCs.<br><br>*→ 1 Introduction*
**The literature search was systematic**	✓ The following <u>keywords</u> were included: (("alcohol*" OR "tobacco" OR "nicotine" OR "smok*" OR "cannabi*" OR "marijuana" OR "thc" OR "cocaine" OR "amphetamine*" OR "methamphetamine" OR "stimulant*" OR "ecstasy" OR "mdma" OR "opiate*" OR "morphine" OR "heroin" OR "benzodiazepine*" OR "analgetic*" OR "hallucinogen*" OR "lsd" OR "ketamine" OR "fentanyl" OR "drug*" OR "substance") AND ("functional magnetic resonance imaging" OR "fmri" OR "functional MRI") AND ("response inhibition" OR "go nogo" OR "stop signal"))<br><br><u>Databases</u>:<br>PsycINFO, PsycARTICLES, Medline Complete, CINAHL Complete and Psychology, Behavioral Sciences Collection, and PubMed<br><br>*→ 2 Methods: "2.1. Search strategy and data acquisition"*
**Detailed inclusion and exclusion criteria are included**	✓ We formulated in-/exclusion criteria a priori based on state-of-the-art recommendations for neuroimaging meta-analyses.<br><br>*→ 2 Methods: "2.1. Search strategy and data acquisition"*
**Sample overlap was taken into account**	✓ Overall, we did not suspect sample overlap in included studies after checking reference lists, scanning parameters, and sample characteristics of included studies. One study reported two eligible experiments using the same sample, so we pooled these foci into one experiment, because otherwise it would violate sample independency assumptions.

	→ *3 Results (first paragraph)*
**All experiments use the same search coverage (state how brain coverage is assessed and how small volume corrections and conjunctions are taken into account)**	✓ All experiments must contain whole brain analyses only. Whole brain coverage was assessed by scanning parameters reported in original studies and compared to average brain measures provided by Müller et al. (2018).<br><br>→ *2 Methods: "2.1. Search strategy and data acquisition"*
**Studies are converted to a common reference space**	✓ Coordinates reported in Talairach reference space were transformed into MNI space by applying the Lancaster transform icbm2tal implemented in GingerALE software.<br><br>→*2 Methods: "2.2 Activation likelihood estimation (ALE) meta-analysis"*
**Data extraction have been conducted by two investigators (ideal case) or double checked by the same investigator (state how doublechecking was performed)**	✓ As follows:<br>MF checked inclusion criteria<br>MF extracted and double checked coordinates<br>MF extracted sample characteristics<br>SN replicated the systematic literature search<br>MM supervised all data
**The paper includes a table with at least the references, basic study description (e. g. for fMRI tasks: stimuli), contrasts and basic sample descriptions (e.g., size, mean age and gender distribution, specific characteristics) of the included studies, source of information (e.g., contact with authors), reference space**	✓ The table contains demographic information (sample size, mean age, gender distribution, substance class, illness duration, abstinence time) and design related information (NoGo/Stop signal ratio, contrasts, correction methods) and number of foci obtained through original studies<br><br>→ *3 Results: "Table 1"*
**The study protocol was previously registered and all analyses planned beforehand, including the methods and parameters used for inference, correction for multiple testing, etc.**	✓ We pre-registered our systematic review and meta-analysis on PROSPERO (CRD42022374754) before starting the systematic literature research. Statistical analyses of ALE were formulated beforehand and adhere to recommendations performing ALE meta-

	analyses. We added a post-hoc random-effects meta-analyses, which is clearly stated as a post-hoc procedure.<br><br>→ *2 Methods: “2.1. Search strategy and data acquisition”; “2.6. Post-hoc exploratory meta-analysis of behavioural data*
**The meta-analysis includes diagnostics**	✓ Diagnostics include number of studies contributing, allocation of experiments that contribute to the convergence cluster, and displaying an overview about original study characteristics as well as robustness measures of findings<br><br>→ *3 Results: “First paragraph”; “Table 2”;*

Table S3 MACM results of significant co-activation with the resulting convergence cluster

		Peak voxel coordinate (MNI)					
Cluster #	Anatomical Label[a]	x	y	z	BA	Cluster Size (mm³)	ALE * (10^{-2})[b]
1	L Cerebrum.Limbic Lobe.Cingulate Gyrus.	0	30	38	32	14.560	8.96
	L Cerebrum.Frontal Lobe.Medial Frontal Gyrus.	-2	18	46	6		8.64
	L Cerebrum.Limbic Lobe.Cingulate Gyrus.	2	34	24	32		5.02
2	R Cerebrum.Sub-lobar.Insula	32	24	-12	47	12.608	34.92
3	L Cerebrum.Sub-lobar.Claustrum	-30	22	-8	-	11.848	10.79
	L Cerebrum.Sub-lobar.Insula	-44	18	-6	13		6.90
	L Cerebrum.Frontal Lobe.Precentral Gyrus.	-56	8	8	44		2.99
4	R Cerebrum.Sub-lobar.Thalamus.Medial Dorsal Nucleus.	6	-18	8	-	7.480	5.24
	R Cerebrum.Sub-lobar.Thalamus.Ventral Lateral Nucleus.	12	-10	2	-		5.08
	R Cerebrum.Sub-lobar.Caudate Head.	10	8	0	-		4.26

	R Cerebrum.Sub-lobar.Thalamus.	12	2	2	-		4.10
	R Cerebrum.Sub-lobar.Thalamus.	8	-18	-4	-		3.66
	R Brainstem.Midbrain.Mammillary Body.	4	-14	-12	-		3.31
	L Brainstem.Midbrain.Mammillary Body.	0	-14	-14	-		3.15
	R Cerebrum.Sub-lobar.Caudate Body.	12	6	14	-		2.88
5	L Cerebrum.Frontal Lobe.Inferior Frontal Gyrus.	-46	8	28	9	4.624	5.31
	L Cerebrum.Frontal Lobe.Precentral Gyrus.	-50	22	28	6		5.18
	L Cerebrum.Frontal Lobe.Middle Frontal Gyrus.	-50	22	28	9		3.62
6	R Cerebrum.Frontal Lobe.Inferior Frontal Gyrus.	46	12	28	9	3.352	3.69
	R Cerebrum.Frontal Lobe.Middle Frontal Gyrus.	48	8	44	6		3.60
	R Cerebrum.Frontal Lobe. Middle Frontal Gyrus.	46	32	18	46		3.59
	R Cerebrum.Frontal Lobe. Inferior Frontal Gyrus.	52	16	28	9		3.19
	R Cerebrum.Frontal Lobe. Middle Frontal Gyrus.	44	44	20	10		2.68
7	L Cerebrum.Sub-Lobar.Lentiform Nucleus.Medial Globus Pallidus.	-12	0	-8	-	2.880	4.47

	L Cerebrum.Sub-Lobar.Lentiform Nucleus.	-12	10	-6	-		4.25
	L Cerebrum.Sub-Lobar.Thalamus.Ventral Lateral Nucleus.	-10	-10	2	-		3.40
8	L Cerebrum.Parietal Lobe.Inferior Parietal Lobule.	-32	-56	52	7	2.744	4.12
	L Cerebrum.Parietal Lobe.Inferior Parietal Lobule.	-42	-50	52	40		3.68
	L Cerebrum.Parietal Lobe.Precuneus.	-26	-68	38	7		2.98

Note. BA, brodman area; L, left; R, right; x,y,z coordinates are displayed in MNI reference space.

[a] Anatomical labelling refers to MNI atlas (nearest grey matter) of peaking coordinate.

[b] Maximum ALE value of the cluster.
